# Circadian rhythm of brain‐derived neurotrophic factor in serum and plasma

**DOI:** 10.1113/EP091671

**Published:** 2024-08-06

**Authors:** Maren Ehrhardt, Stefanie Schreiber, Yves Duderstadt, Rüdiger Braun‐Dullaeus, Katrin Borucki, Tanja Brigadski, Notger G. Müller, Volkmar Leßmann, Patrick Müller

**Affiliations:** ^1^ Division of Cardiology and Angiology University Hospital Magdeburg Magdeburg Germany; ^2^ German Center for Neurodegenerative Diseases (DZNE) Magdeburg Germany; ^3^ Center for Intervention and Research on Adaptive and Maladaptive Brain Circuits Underlying Mental Health (C‐I‐R‐C) Magdeburg Germany; ^4^ Division of Neurology University Hospital Magdeburg Magdeburg Germany; ^5^ Department of Neurology, Medical Faculty Heinrich Heine University Düsseldorf Germany; ^6^ Institute of Sport Science Otto‐von‐Guericke University Magdeburg Germany; ^7^ Institute of Clinical Chemistry and Pathobiochemistry Otto‐von‐Guericke University Magdeburg Germany; ^8^ Institute of Physiology Otto‐von‐Guericke University Magdeburg Germany; ^9^ Department of Informatics and Microsystems Technology University of Applied Sciences Kaiserslautern Zweibrücken Germany; ^10^ Degenerative and Chronic Diseases, Faculty of Health Sciences Brandenburg University of Potsdam Potsdam Germany; ^11^ German Center for Mental Health (DZPG) Magdeburg Germany; ^12^ Center for Behavioural Brain Sciences (CBBS) Magdeburg Germany

**Keywords:** biomarker, brain‐derived neurotrophic factor, circadian rhythm, sleep

## Abstract

The neurotrophic growth factor brain‐derived neurotrophic factor (BDNF) plays a crucial role in various neurodegenerative and psychiatric diseases, such as Alzheimer's disease, schizophrenia and depression. BDNF has been proposed as a potential biomarker for diagnosis, prognosis and monitoring therapy. Understanding the factors influencing BDNF levels and whether they follow a circadian rhythm is essential for interpreting fluctuations in BDNF measurements. We aimed to investigate the circadian rhythm of BDNF by collecting multiple peripheral venous blood samples from young, healthy male participants at 12 different time points over 24 h. In addition, vital parameters, cortisol and insulin like growth factor 1 (IGF1) were measured to explore potential regulatory mechanisms, interfering variables and their correlations with BDNF concentration. The findings revealed that plasma BDNF did not exhibit any significant fluctuations over 24 h, suggesting the absence of a circadian rhythm. However, serum BDNF levels decreased during sleep. Furthermore, serum BDNF showed a positive correlation with heart rate but a negative correlation with IGF1. No significant correlation was observed between cortisol and BDNF or IGF1. Although plasma BDNF suggests steady‐state conditions, the decline of serum BDNF during the nocturnal period could be attributed to physical inactivity and associated with reduced haemodynamic blood flow (heart rate reduction during sleep). The type of sample collection (peripheral venous cannula vs. blood sampling using a butterfly system) does not significantly affect the measured BDNF levels. The sample collection during the day did not significantly affect BDNF analysis, emphasizing the importance of considering activity levels rather than timing when designing standardized protocols for BDNF assessments.

## Abstract (highlights)

- **What is the central question of this study?**
  How does the circadian rhythm potentially influence peripheral brain‐derived neurotrophic factor (BDNF) levels, and what has to be taken into consideration when interpretating BDNF levels in serum or plasma as potential biomarkers in neurodegenerative or psychiatric diseases?
- **What is the main finding and its importance?**
  Although plasma BDNF levels remain relatively stable over 24 h, serum BDNF exhibits a significant nocturnal decline, potentially linked to physical inactivity during sleep. Understanding this rhythm and the factors influencing BDNF levels is crucial for accurate interpretation of BDNF measurements, emphasizing the need to consider activity levels in standardized protocols for BDNF assessments.

## 1 INTRODUCTION

Brain‐derived neurotrophic factor (BDNF) was discovered in the 1980s (Barde et al., 1982) as the second member of the neurotrophin (NT) protein family. It can be detected in various regions of the CNS, with the highest expression found in the cerebral cortex and hippocampus (Edelmann et al., 2014), but is also found in various peripheral tissues (Cartwright et al., 1994), organs (Katoh‐Semba et al., 1997) and blood cells, including platelets/thrombocytes (Fujimura et al., 2002) and lymphocytes (Kerschensteiner et al., 1999). Release of BDNF into extracellular space or bloodstream takes place through exocytosis via either the constitutive or the regulated pathway of secretion, which is triggered by cytoplasmic Ca^2+^ elevation (Brigadski & Leßmann, 2020). BDNF plays a crucial role in long‐term potentiation in all cortical brain areas, the hippocampus, the amygdala and other subcortical brain regions, thereby mediating learning and memory processes (Gottmann et al., 2009; Lu et al., 2014; Park & Poo, 2013).

Differences in cerebral or peripheral concentrations of BDNF have been observed in various neurodegenerative and psychiatric disorders, such as Alzheimer's disease (Angelucci, 2009; Laske et al., 2006), major depressive disorder (Karege et al., 2002) and schizophrenia (Hashimoto et al., 2005). BDNF release is also associated with numerous normal physiological and pathophysiological processes, such as maintaining vascular and endothelial stability and regulating inflammation, thus playing a role in non‐neurological diseases (Boyuk et al., 2014; Donovan et al., 2000; Freeman et al., 2017; Stoll et al., 2012). Therefore, it has been suggested that measurements of BDNF concentrations might have the potential to assess the severity of various diseases (Laske & Eschweiler, 2006). However, in order to use BDNF as a potential biomarker and facilitate early clinical decisions, fundamental aspects require clarification, including understanding the influence of various factors that might lead to peripheral BDNF fluctuations (Balietti et al., 2018). For example, age, sex (Lommatzsch et al., 2005), hormonal status (Begliuomini et al., 2007; Verhovshek et al., 2010), exercise (Müller et al., 2017, 2020; Rehfeld et al., 2018), physical activity (Cho et al., 2012; Rasmussen et al., 2009) and conditions such as hypoxia (Becke et al., 2018) have been shown to influence circulating levels of BDNF. The regulation of hippocampal BDNF is attributed to factors such as insulin like growth factor 1 (IGF1), which impacts synaptic and cognitive plasticity through interactions in processes induced by physical training (Arazi et al., 2021; Ding et al., 2006). Sensory stimuli, such as alterations in illumination and disturbances in the light–dark and sleep–wake cycles (Castrén et al., 1992; Hamatake et al., 2011; Molendijk et al., 2012), psychotropic medications (Huopaniemi et al., 2004; Lee & Kim, 2008; Leyhe et al., 2008) and non‐psychotropic medications (Patil et al., 2014; Zhang et al., 2017), in addition to lifestyle habits, such as smoking (Bhang et al., 2010), alcohol consumption (Logrip et al., 2009), ketamine abuse (Woelfer et al., 2020) and psychosocial stress (Rasmusson et al., 2002; Roceri et al., 2004), can affect BDNF levels. Understanding the factors that influence the concentration of BDNF is crucial for establishing it as a marker for diagnostic purposes and to be able to assess and evaluate study findings.

Another important control variable for blood parameters is the circadian rhythm. Circadian oscillation describes the rhythmic patterns that occur with a specific frequency over the course of a day. In the CNS, the suprachiasmatic nucleus of the anterior hypothalamus acts as the primary biological clock and endogenous oscillator, coordinating the activities of a multicellular organism (Hastings, 1997). Various factors can disrupt the circadian system, including shift work, jet lag, artificial light, irregular sleep and eating schedules, and even chronic social stress. Proper coordination of the entire circadian system is crucial for maintaining overall health and well‐being (Rosenwasser & Turek, 2015). *BDNF* and *trkB* mRNA concentrations in the frontal cortex and hippocampus of rats during a 24 h cycle showed diurnal fluctuations (Schaaf et al., 2000). These could be attributable to endogenous rhythmicity or related to fluctuations in activity levels. It was found that BDNF induction takes place simultaneously with the onset of the activity period during the dark cycle (Berchtold et al., 1999; Bova et al., 1998) and that *BDNF* mRNA is elevated in the visual cortex simultaneously with the opening of the eyes (Castrén et al., 1992). Alterations in the basal *BDNF* mRNA expression within the hippocampus were linked to the plasma concentration of corticosteroids (Schaaf et al., 2000). The changes observed in BDNF levels within non‐visual parts of the brain indicate that hormones or motor activity plays a role in plasticity mediated by BDNF (Pollock et al., 2001).

Initial studies exploring the daily rhythm of BDNF in humans found that its plasma levels were highest in the morning (8:00 a.m.) and decreased throughout the day, reaching a minimum with the last sampling at midnight (Begliuomini et al., 2008). Other studies have also shown similar circadian secretion patterns (Choi et al., 2011; Piccinni et al., 2008; Pluchino et al., 2009). The diurnal changes in BDNF levels, similar to those of cortisol, suggest a potential synergistic role between BDNF and glucocorticoids in homeostasis of cerebral functions in humans (Begliuomini et al., 2008). However, other studies have reported no diurnal variation in BDNF levels in women and attributed this phenomenon to the influence of varying sex hormones and disparities in the regulation of the hypothalamic–pituitary–adrenal axis between sexes (Choi et al., 2011; Piccinni et al., 2008). In another study, a constant routine protocol was implemented, and plasma BDNF levels varied widely throughout the day, with peak levels occurring at different time points (Cain et al., 2017).

The aim of the present study was to investigate nocturnal BDNF concentrations in addition to influencing factors that might determine whether BDNF serum or plasma levels exhibit a circadian rhythm or another pattern of rhythmic change. To accomplish this objective, serum and plasma samples were collected more frequently at 12 specific testing time points spanning a 24 h duration while maintaining natural sleep–wake rhythm and light–dark cycle. Concurrently, participants adhered strictly to a 24 h protocol designed to emulate their individual ‘natural rhythm’ closely. Moreover, comprehensive data on factors affecting BDNF levels, such as physical activity, meals, sleep–wake patterns and hormones, such as cortisol and IGF1, were gathered to enable a thorough analysis and evaluation of BDNF fluctuations.

## 2 MATERIALS AND METHODS

### 2.1 Ethical approval

The study was designed as a prospective, controlled study investigating circadian rhythm and received approval from the ethics committee at the Otto‐von‐Guericke University Magdeburg (Germany, ethical approval number 105/20). All participants signed a written informed consent form prior to participation. The studies adhered to the standards outlined in the most recent version of the *Declaration of Helsinki*, except for registration in a database. Recruitment of participants was conducted through advertisements at the Medical Faculty and public notices.

### 2.2 Participants

To ensure a high level of homogeneity within the study group and to minimize other potentially influencing factors, such as hormonal status, only male participants (*n* = 10) were recruited and examined from 7 November 2020 to 6 December 2020 at the German Center for Neurodegenerative Diseases (DZNE) Magdeburg, Germany. The age of participants ranged from 18 to 31 years (mean ± SD = 25.3 ± 3.59 years) and the body mass index from 19.79 to 28.4 kg/m^2^ (mean ± SD = 22.72 ± 2.55 kg/m^2^). The participants were required to have no history of mental or physical diseases (of a neurological, psychogenic, musculoskeletal, cardiorespiratory or systemic nature). Additionally, participants who were smokers, users of illegal substances, consumers of medications with CNS effects, recent travellers across time zones in the past 3 months, individuals experiencing sleep–wake cycle disturbances in the week prior to the study or those regularly working night shifts were excluded from the study. Throughout the experiment, the physical activity of participants was monitored using a GPS smartwatch, and multiple routine laboratory tests, including a complete blood count and electrolytes, were performed, which revealed no abnormalities.

### 2.3 Protocol

**FIGURE 1:**
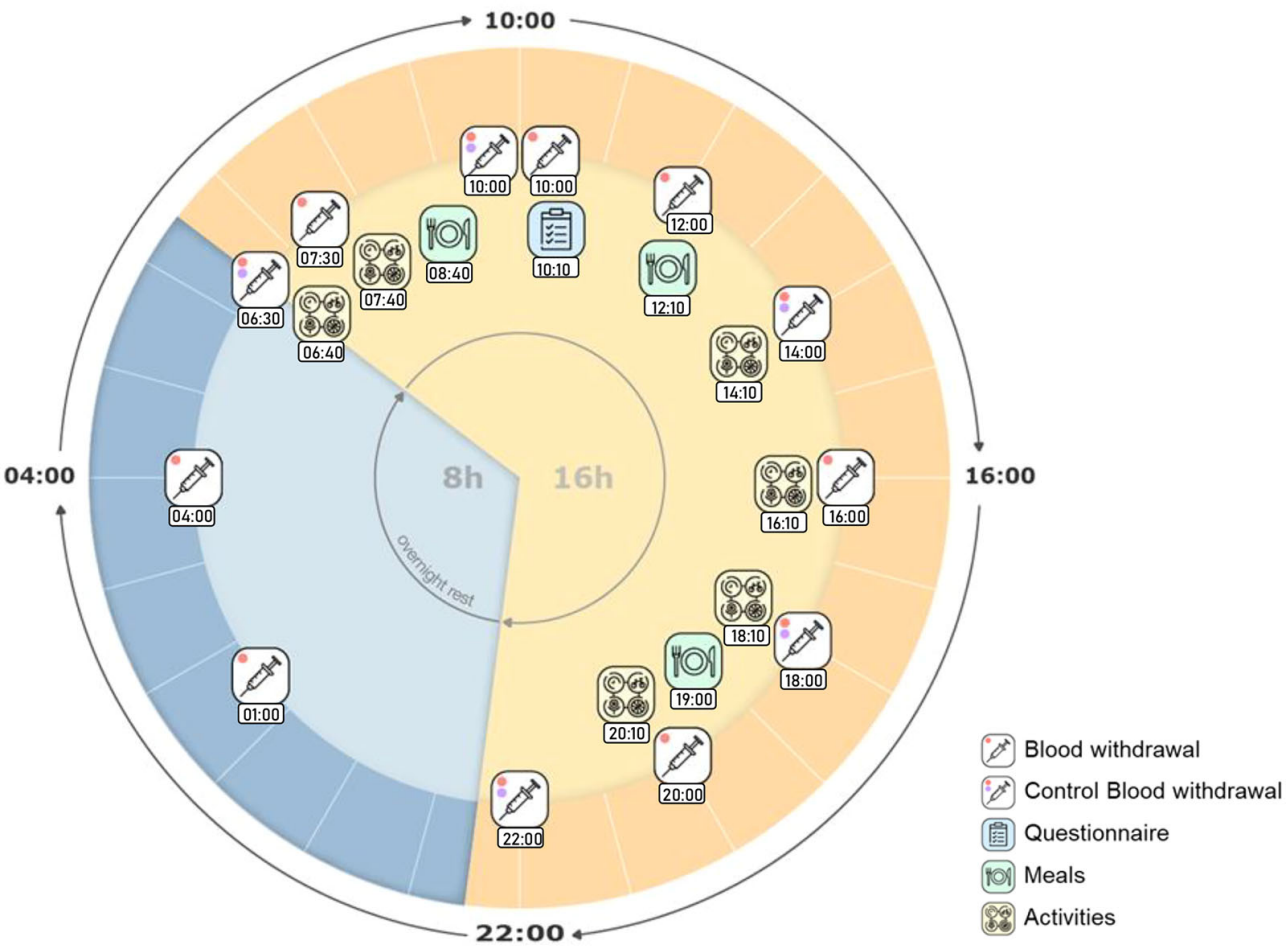
Overview of the experiment, illustrating the complete 24 h period, including all blood samplings, activities (14:10–16:00 h: walking outside; 16:10–17:00 h: playing a boardgame; 17:00–18:00 h: cycling inside; 18:10–19:00 h: reading; 20:10–22:00 h: watching a movie; 06:40–07:30 h: getting ready; 07:30–08:30 h: walking outside), overnight rest and meals.

Subject‐specific information (age, sex, height and weight) was recorded, and participants were asked about their health status. Each subject was equipped with a GPS smartwatch (vivoactive 4, Garmin, 010‐02174‐02) and a peripheral venous cannula (BD Venflon Pro, 18 gauge, BD‐75118, NJ, USA) was inserted into the antecubital region for blood collection purposes. To investigate whether a diurnal rhythm of plasma and serum BDNF exists and to analyse potential correlations or influences of IGF1, complete blood count and electrolytes, blood samples were obtained at 12 time points [10:00 h (*t*_1_), 12:00 h (*t*_2_), 14:00 h (*t*_3_), 16:00 h (*t*_4_), 18:00 h (*t*_5_), 20:00 h (*t*_6_), 22:00 h (*t*_7_), 01:00 h (*t*_8_), 04:00 h (*t*_9_), 06:30 h (*t*_10_), 07:30 h (*t*_11_) and 10:00 h (*t*_12_) the next day] from each participant over a 24 h period. At five test time points (*t*_3_, *t*_5_, *t*_7_, *t*_10_ and *t*_12_), an additional venipuncture (Safety‐Multifly cannula 21 gauge, Sarstedt‐85.1638.235, Karlsruhe, Germany) was performed on the other arm to collect control samples for measurement of serum and plasma BDNF, aiming to identify any possible influence resulting from the peripheral venous cannula cannula used for sampling. Furthermore, cortisol levels were determined by analysis of saliva samples collected using salivettes (Cortisol Salivette, Sarstedt‐51.1534.500, Nümbrecht). To ensure consistent experimental conditions and minimize potential influencing factors, the sampling process was conducted according to a daily protocol that specified daily activities of participants, including walking outside two times a day and indoor cycling for 45 min, post‐waking behaviour, meal timings (three per day), daily water intake (1.5 L/day) and times of resting at night (Figure 1).

### 2.4 BDNF assay

Blood samples were obtained using a standardized protocol. Plasma samples for analysis of BDNF concentration were collected using lithium heparin tubes (Vacutainer heparin tube, BD‐368886, Heidelberg, Germany), carefully swirled upside down 10 times, then stored on ice for 7 min. Afterwards, the samples were centrifuged at 2000*g* and 20°C for 15 min to separate the plasma, which was then stored at −80°C for further analysis. Likewise, BDNF serum samples (Vacutainer SST II Advance tube, BD‐366566) were gently swirled upside down 10 times, then incubated at room temperature for 30 min to allow clot formation. Subsequently, the samples were centrifuged at 2000*g* and 20°C for 15 min and also stored at −80°C.

The analysis of BDNF was performed by the Institute of Physiology at the University of Magdeburg, Germany. Quantitative determination of BDNF levels was carried out using an enzyme‐linked immunosorbent assay (ELISA) kit, following the instructions provided by the manufacturer [Duoset ELISA Development Kit, R&D Systems Europe, Wiesbaden, Germany; catalogue number of the kit, DY248; range, 11.7–750 mg/mL; specificity, no cross‐reactivity or interference with Nerve growth factor, Glial cell line‐derived neurothrophic factor Neurotrophin‐3 or Neurotrophin‐4 according to the manufacturer's instructions; tested intra‐assay coefficient of variation for serum: minimum–maximum, 0–5, median (95% confidence interval of median): 0.9 (0.1–2.9); tested intra‐assay coefficient of variation for plasma: coefficient of variation, 0–9; median (95% confidence interval of median), 1.0 (0.1–4.5); tested interassay coefficients: standard, minimum–maximum, 5.2–10.5, median (95% confidence interval of median), 8.6 (5.3–10.3)]. Both plasma and serum samples from one subject collected via blood sampling were analysed on one plate. Furthermore, serum BDNF from one subject collected via blood sampling and through a venous indwelling cannula were also analysed on one plate. For the quantification of BDNF, plasma samples were diluted 1:8 and serum samples 1:128. Dilution linearity was obtained in the range of 1:6, 1:8, 1:10 and 1:12 for plasma samples (coefficient of variation: minimum–maximum, 2.2–5.8) and in the range of 1:64, 1:80, 1:100, 1:128, 1:150 and 1:180 for serum samples (coefficient of variation: minimum–maximum, 0.6–4.8).

### 2.5 Cortisol assay

Cortisol levels were assessed at *t*_1_, *t*_3_, *t*_5_, *t*_8_, *t*_9_, *t*_10_ and *t*_12_ using salivettes (Cortisol‐Salivette, Sarstedt‐51.1534.500, Nümbrecht). After collection, the samples were stored at 5°C. After each experimental run, the salivettes were centrifuged at 1000*g* for 2 min to separate the saliva. The clear supernatant was then used for the immunological in vitro assay (Elecsys Cortisol II, Roche, Mannheim, Germany) on an immunoassay system (cobas e, Roche).

### 2.6 IGF1 assay

The collected serum samples (*t*_1_, *t*_3_, *t*_5_, *t*_7_, *t*_10_ and *t*_12_) were stored at room temperature for 7 min, then centrifuged at 2000*g* and 20°C for 15 min. The supernatant was collected and stored at −20°C. Subsequent processing was carried out by the Institute of Clinical Chemistry and Pathobiochemistry at the University of Magdeburg, Germany 2 months after the last sample collection. After separation of the binding protein from IGF1, the quantitative determination of bound and unbound IGF1 was performed using a one‐step sandwich chemiluminescence immunoassay and a LIAISON analyser (DiaSorin, Saluggia, Italy).

### 2.7 Assay of complete blood count and electrolytes

The collected samples were stored at room temperature for 1 h before being processed by the Institute of Clinical Chemistry and Pathobiochemistry at the University of Magdeburg, Germany. A complete blood count was performed on an XN‐10 (Sysmex, Norderstedt, Germany). In addition, plasma was collected using a lithium‐heparin tube to obtain information about electrolytes. The quantitative determination of calcium in plasma was performed using a photometric assay (Calcium Gen.2 with cobas c System, Roche/Hitachi, Mannheim, Germany). The levels of sodium, calcium and chloride were determined using ion‐selective electrodes (ISE indirect Na‐K‐Cl for Gen.2 with cobas c system, Roche/Hitachi).

### 2.8 Pittsburgh sleep quality index

To assess the participants’ sleep as a potential influencing variable, the Pittsburgh sleep quality index self‐assessment questionnaire was completed. The questionnaire was evaluated according to scoring instructions provided by Buysse et al. (1989).

### 2.9 Parameters and statistical analysis

For statistical analysis of the data, IBM SPSS Statistics v.27 software was used. Normal distribution of all variables was tested using the Kolmogorov–Smirnov test and graphically, including *Q–Q* plots. To investigate the influence of sampling time on the collected parameters, single‐factor ANOVAs with repeated measures were performed for plasma and serum BDNF, cortisol and IGF1. Additional *post hoc* tests were conducted to analyse intra‐ and inter‐individual differences. Linear Pearson correlation analysis was examined to assess the interrelationships between BDNF, cortisol, IGF1 and the heart rate of participants.

## 3 RESULTS

### 3.1 Smartwatch

**FIGURE 2:**
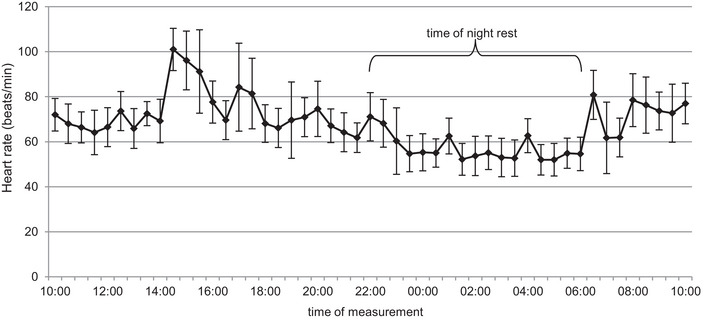
Mean ± SD heart rate measured by Smartwatch during the experiment. *Post hoc* testing showed a decrease during the night from 16:00 to 00:00 h (*P* = 0.002) and increase when starting activity, 14:00 and 14:30 h (*P* > 0.001).

The average effective sleep duration was measured (*n* = 10; mean sleep duration = 7.34 ± 0.69 h). All participants exhibited physiological heart and respiratory rates in relationship to standard values that increased during physical activity and decreased during rest. Depending on the activity level, heart rate ranged from 42 to 126 beats/min and respiratory rate from 10 to 21 breaths/min. The heart rate showed a decrease at night (mean heart rate awake = 73.23 ± 4.97 beats/min; mean heart rate asleep = 55.38 ± 7.14 beats/min); a one‐way ANOVA revealed significant differences between the heart rate at different measurement time points (*n* = 482, *F* = 1.388, *P* ≤ 0.001), a *post hoc* test showed a decrease during the night (e.g., 16:00 to 00:00 h, *P* = 0.002) and an increase when starting activity (14:00 and 14:30 h, *P* > 0.001; Figure 2).

### 3.2 Blood samples

Analysis of the complete blood count showed no pathological abnormalities in any of the participants. The levels of sodium, calcium, chloride and potassium were within the reference ranges. There was no effect of blood sampling (in total, 257 mL) on the platelet count (*F* = 1.308, *P* = 0.277, η^2^ = 0.127) or the erythrocyte count (*F* = 2.100, *P* = 0.083, η^2^ = 0.189).

### 3.3 BDNF

**FIGURE 3:**
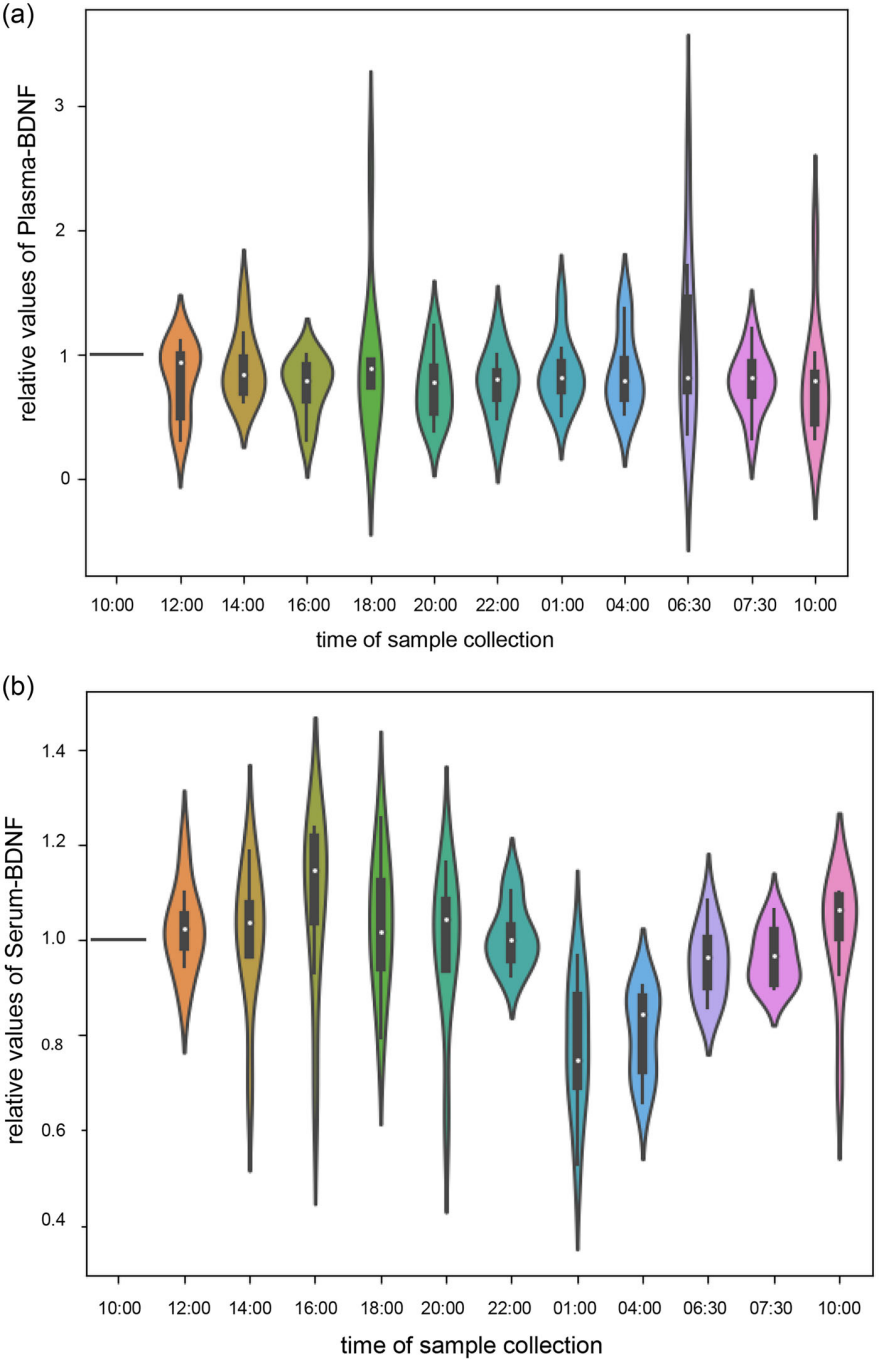
Violin plots of relative values for plasma brain‐derived neurotrophic factor (BDNF) (a) and serum BDNF (b) over 24 h. *Post hoc* testing showed that serum BDNF decreases during the night at *t*_8_ and *t*_9_, with a significant difference between *t*_8_ and *t*_1_ (*P* = 0.050), *t*_2_ (*P *= 0.050), *t*_4_ (*P *≤ 0.001) and *t*_10_ (*P* = 0.023) and between *t*_9_ and *t*_1_ (*P* = 0.006), *t*_2_ (*P* = 0.007), *t*_4_ (*P* = 0.015), *t*_5_ (*P* = 0.026), *t*_7_ (*P* = 0.036), *t*_10_ (*P* ≤ 0.001) and *t*_11_ (*P* = 0.014).

A repeated‐measures ANOVA revealed no significant circadian changes in plasma BDNF (*F* = 1.639, *P* = 0.099, η^2^ = 0.154) but significant changes in serum BDNF levels (*F* = 7.769, *P* < 0.001, η^2^ = 0.463). *Post hoc* testing showed that serum BDNF decreases during the night at *t*_8_ and *t*_9_, with a significant difference for *t*_8_ and *t*_1_ (*P* = 0.050), *t*_2_ (*P *= 0.050), *t*_4_ (*P *≤ 0.001) and *t*_10_ (*P* = 0.023), in addition to *t*_9_ and *t*_1_ (*P* = 0.006), *t*_2_ (*P* = 0.007), *t*_4_ (*P* = 0.015), *t*_5_ (*P* = 0.026), *t*_7_ (*P* = 0.036), *t*_10_ (*P* ≤ 0.001) and *t*_11_ (*P* = 0.014). To eliminate inter‐individual variability, serum and plasma BDNF levels are presented relative to the initial value for the 24 h in a violin plot (Figure 3).

To compare the impact of venipuncture using a butterfly system and a peripheral indwelling cannula on sample collection, plasma and serum BDNF samples were additionally taken and compared at five time points (*t*_3_, *t*_5_, *t*_7_, *t*_10_ and *t*_12_). The results were analysed using Student's paired *t*‐test, which revealed no significant difference in plasma (*P* = 0.881) and serum (*P* = 0.109) BDNF levels. Therefore, the method of sample collection does not have a statistically significant effect on the results.

### 3.4 Correlation analysis of BDNF and heart rate

**FIGURE 4:**
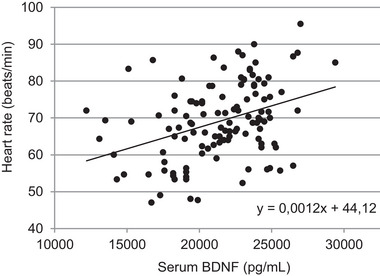
Moderate positive correlation (*r* = 0.369, *P* ≤ 0.001) between serum brain‐derived neurotrophic factor (BDNF) and heart rate (*t*_1_–*t*_12_).

**FIGURE 5:**
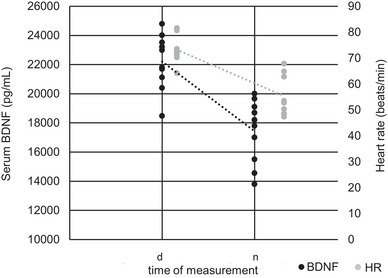
Comparison of serum BDNF level and HR during the day (d: mean values of heart rate and BDNF awake) and night rest (n: mean values of heart rate and BDNF asleep). Abbreviations: BDNF, brain‐derived neurotrophic factor; HR, heart rate.

A Pearson correlation analysis between serum BDNF and heart rate at *t*_1_–*t*_12_ revealed a moderate positive correlation (*r* = 0.369, *P* = < 0.001; Table 1; Figure 4). Serum BDNF and heart rate exhibited a decrease at night (Figure 5). However, the correlation analysis assessing the delta‐changes in BDNF and heart rate between mean values during the day (d: mean values of heart rate and BDNF awake) and at night (n; mean values of heart rate and BDNF asleep) did not yield statistical significance (*r* = −0.092, *P* = 0.800).

**TABLE 1:** Pearson correlation analysis for cortisol, serum BDNF, heart rate and IGF1, two‐sided significance.

	Cortisol (nmol/L)	Serum BDNF (pg/mL)	IGF1 (ng/mL)	Heart rate (beats/min)
Cortisol (nmol/L)	1	−0.021	0.213	0.068
Serum BDNF (pg/mL)		1	−0.359**	0.369***
IGF1 (ng/mL)			1	0.131
Heart rate (beats/min)				1

** = *p* < 0.01; *** = *p* < 0.001.

### 3.5 Saliva samples

**FIGURE 6:**
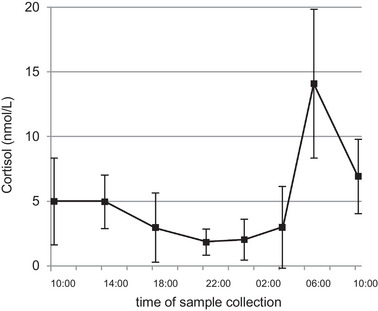
Mean values (±SD) measured for cortisol over 24 h. *Post hoc* testing showed a significant difference between *t*_10_ and *t*_3_ (*P* = 0.031), *t*_5_ (*P* = 0.016), *t*_7_ (*P* = 0.004), *t*_8_ (*P* = 0.006) and *t*_9_ (*P* = 0.005) (*t*_3_ = 14:00 h; *t*_5_ = 18:00 h; *t*_7_ = 22:00 h; *t*_8_ = 01:00 h; *t*_9_ = 04:00 h; *t*_10_ = 06:30 h).

Cortisol levels showed a peak at 06:30 h (mean cortisol at *t*_10_ = 14.09 ± 5.76 nmol/L), with a gradual decrease throughout the day, reaching the lowest level at 22:00 h (mean cortisol at *t*_7_ = 1.83 ± 1 nmol/L; Figure 6). A repeated‐measures ANOVA revealed a significant change in circadian cortisol levels (*F* = 16.740, *P* ≤ 0.001, η^2^ = 0.650). *Post hoc* tests showed an increase at *t*_10_, with a significant difference for *t*_10_ and *t*_3_ (*P* = 0.031), *t*_5_ (*P* = 0.016), *t*_7_ (*P* = 0.004), *t*_8_ (*P* = 0.006) and *t*_9_ (*P* = 0.005). We did not observe a general significant association between cortisol and serum BDNF (*r* = −0.021, *P* = 0.851) or between cortisol and IGF1 levels (*r* = −0.213, *P* = 0.103; Table 1).

### 3.6 IGF1

**FIGURE 7:**
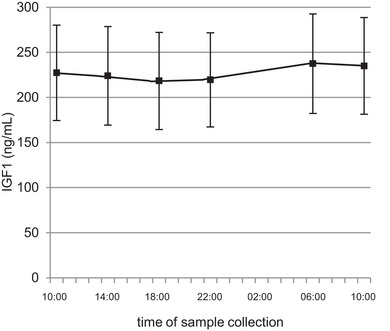
Mean values (± SD) measured for insulin like growth factor 1 (IGF1) in serum over 24 h. *Post hoc* testing showed a significant difference between *t*_10_ and *t*_5_ (*P* ≤ 0.001), *t*_7_ (*P* = 0.002) and *t*_12_ and between *t*_5_ (*P* = 0.024) and *t*_7_ (*P* = 0.039) (*t*_5_ = 18:00 h; *t*_7_ = 22:00 h; *t*_10_= 06:30 h; *t*_12_ = 10:00 h the next day).

**FIGURE 8:**
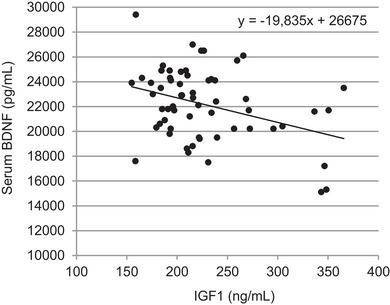
Moderate negative correlation (*r* = −0.359, *P* = 0.005) between serum brain‐derived neurotrophic factor (BDNF) and insulin like growth factor 1 (IGF1) (*t*_1_–*t*_12_).

A repeated‐measures ANOVA was conducted, revealing a significant increase of IGF1 in the morning (*F* = 7.517, *P* ≤ 0.001, η^2^ = 0.455; Figure 7). *Post hoc* testing showed an increase at *t*_10_ and *t*_12_, with significant differences between *t*_10_ and *t*_5_ (*P* ≤ 0.001) and *t*_7_ (*P* = 0.002) and between *t*_12_ and *t*_5_ (*P* = 0.024) and *t*_7_ (*P* = 0.039). A Pearson correlation analysis revealed a moderate negative correlation for the results of IGF1 and serum BDNF (*r* = −0.359, *P* = 0.005; Table 1; Figure 8).

### 3.7 Pittsburgh sleep quality index

The Pittsburgh sleep quality index assessment (Buysse et al., 1989) identified disturbed sleep in 3 of 10 participants (participants 4, 5 and 9).

## 4 DISCUSSION

The objective of the study was to investigate the diurnal dynamics of BDNF and analyse potential circadian rhythms or cyclic patterns. At the same time, factors that could affect these changes were either minimized or documented. Previous studies suggested a potential endogenous rhythm of plasma BDNF in humans (Begliuomini et al., 2008; Cain et al., 2017; Choi et al., 2011; Piccinni et al., 2008; Pluchino et al., 2009; Woelfer et al., 2020); however, there is currently no conclusive evidence for a circadian rhythm of BDNF. Thus far, no analysis has been conducted over a 24 h period, taking into account the day–night cycle. In our experiment, we did not observe any dynamics in plasma BDNF, precluding any conclusions regarding circadian rhythm, whereas serum BDNF exhibited a nocturnal decline, which could be attributable to a circadian rhythm or associated with haemodynamic changes during sleep or inactivity.

In documenting vital parameters, the Smartwatch showed a physiological response and adaptation to rest and exertion in the context of walking and cycling, in addition to adequate circadian day–night responses (e.g., reduction of heart rate at night). Short‐term sleep disturbances can lead to increased *BDNF* mRNA and protein levels in rat hippocampus. With average sleep duration of 7 h and 20.2 min, none of the participants should have experienced a quantitative sleep deficit. Although the subjective assessment prior to the experiment did not suggest unhealthy sleep in any of the participants, the Pittsburgh sleep quality index showed healthy sleep for only 7 of the 10 participants. However, for the participants with poor sleep (participants 4, 5 and 9), there were no abnormalities, special dynamics or conspicuously low or high scores in the holistic assessment in comparison to the other participants.

The results of the complete blood count and the electrolyte values were unremarkable in all participants. Given that the cell counts and their proportions remained unchanged and showed no dynamics during the 24 h multiple blood collections, no effects on the measured values for BDNF or IGF1 levels were to be expected. Altered electrolyte values can also indicate possible disturbances in physiological processes (Shrimanker & Bhattarai, 2023) or indicate errors in sample collection that can lead to haemolysis, which can be reflected in the blood count as pseudo‐hyperkalaemia (Asirvatham et al., 2013; Saleem et al., 2009). Erythrocyte haemolysis, which is associated simultaneously with platelet activation (Helms et al., 2013), can lead to the release of BDNF because platelets store a large amount of BDNF that is usually released during coagulation (Fujimura et al., 2002). With potassium values in the normal range in all participants, it can, therefore, be assumed that the blood collection had no influence on the data collected in this respect.

The values for BDNF in plasma and serum obtained by immediately preceding venipuncture versus through a peripheral long‐term indwelling cannula showed no significant discrepancy, which supports the assumption that the method of blood sample collection, or the use of a peripheral indwelling venous cannula, is not a confounding factor for the analysis of BDNF. To facilitate data comparison and minimize interassay variability, all BDNF samples collected via a peripheral long‐term indwelling cannula were analysed with the same assay kits. Storage at −80°C for ≤6 months after sample collection is not expected to affect the BDNF content in serum and plasma (Polyakova et al., 2017).

In this controlled study, we did not observe a circadian rhythm of plasma BDNF in male participants. This observation is in contrast to previous findings (Begliuomini et al., 2008; Choi et al., 2011; Piccinni et al., 2008). The lack of a significant decrease of plasma BDNF levels at night could be attributable to the high variance of the data. However, Cain et al. (2017) described wide fluctuations in plasma BDNF levels throughout the day, with the timing of the peak varying greatly between individuals (Cain et al., 2017). This observation is corroborated by our results for plasma BDNF levels. Variability in BDNF results can arise from various factors, including individual differences in physiology, genetics, lifestyle and environmental factors. Although we aimed to control for known sources of variability, other unaccounted factors could contribute to variability in BDNF levels among participants.

Our results show a decrease in serum BDNF during sleep. The nocturnal decrease of serum BDNF might reflect an endogenous circadian cycle, in addition to reduced activity during sleep, which might lead to a reduction of BDNF in the periphery regardless of the time of the day. Previous findings did not show diurnal changes in serum BDNF (Choi et al., 2011; Piccinni et al., 2008). Autonomic control of average heart rate differs between sleep stages (Boudreau et al., 2013). During non‐rapid eye movement (non‐REM) sleep, blood pressure, heart rate and sympathetic nervous system activity in general are lower in comparison to wakefulness (Somers et al., 1993). Pulsatile blood pressure from the heart affects blood circulation and leads to tension and deformation of the vascular wall (Lu & Kassab, 2011) that can increase the BDNF secretion from endothelial cells (Cefis et al., 2022). Platelets are also activated by shear forces, resulting in the release of BDNF (Fujimura et al., 2002). Lower blood pressure and heart rate during sleep are associated with reduced haemodynamic effects on platelet activation. The collected data revealed a correlation between heart rate and serum BDNF levels, a phenomenon that has also been observed in other studies (Currie et al., 2009). The nocturnal decrease in serum BDNF might be attributable either to reduced BDNF secretion from activated platelets during coagulation or to the nocturnal decrease in heart rate. Additionally, Brebbia & Altshuler (1965) demonstrated significant differences in oxygen consumption rates between sleep stages, with higher rates during rapid eye movement (REM) sleep and wakefulness compared with non‐REM stages. They concluded that metabolism decreases during normal sleep and increases again towards morning, potentially impacting BDNF synthesis, release and, subsequently, nocturnal measurable levels.

Moderate activity, meals or time‐of‐day‐dependent light conditions do not seem to influence levels of BDNF directly. Instead, it is important to consider persistent influencing factors in the analysis and subsequent documentation of BDNF levels, such as seasonal variations in the duration of sunlight (Molendijk et al., 2012), hormonal fluctuations dependent on sex (Begliuomini et al., 2007; Pluchino et al., 2009), health‐related behaviours (Bhang et al., 2010; Logrip et al., 2009) including weight and dietary habits (Nakazato et al., 2003), overall fitness (Arazi et al., 2021) and co‐morbidities (Angelucci, 2009; Karege et al., 2002; Scalzo et al., 2010; Takahashi et al., 2000).

The measured values for IGF1 showed an increase in the morning, which can be attributed to the effect of growth hormone on IGF1 synthesis in the liver, which peaks between 02:00 and 04:00 h (Serin & Acar Tek, 2019). In the diurnal profile, IGF1 appeared constant and did not show a directly measurable response to increased activity. Guha et al. (2012) showed that freezing the samples at −20°C for a period of 3 months had no effect on the measured IGF1 levels, hence the time of storage is not likely to be an influencing factor. Studies have shown that strength and endurance interventions can lead to immediate increases in measurable levels of IGF1 and BDNF. However, there are discrepancies in studies investigating the influence on BDNF, which could be attributable to variations in the intensity of the physical activities (Arazi et al., 2021).

The data generated in our 24 h experiment showed a negative correlation between IGF1 levels and BDNF in serum. Samples of IGF1 obtained 10 min after physically more active interventions did not show clear increases or dynamics that would suggest a directly measurable influence in all participants. Based on the moderate increase in heart rate and respiratory rate, it is obvious that all participants experienced an increase in activity, but it was not intense athletic training. This could explain why physical activity‐related increases in serum and plasma BDNF were not detectable. Short‐term physical activity, which does not reach the intensity of athletic training and thus does not lead to increases in IGF1, does not seem to have a clear acute effect on the levels of BDNF in plasma and serum. In practice, this means that moderate physical activity immediately before sample collection (such as low‐ to moderate‐intensity cycling, walking or climbing stairs) does not directly affect BDNF levels in the short term.

Furthermore, both free and carrier protein‐bound IGF1 were measured in the present study. Circulating IGF1 can exist in its unbound form or predominantly bound to carrier proteins (Lewitt et al., 1994). These proteins regulate its flow from the vascular space to tissues, influence IGF1 half‐life and regulate its metabolic clearance. They also modulate the interaction between IGF1 and its receptor, indirectly controlling IGF1 biological activity, in addition to modulating IGF1 levels in target tissues, thereby inhibiting or activating its specific actions (La Garza et al., 2017). To make more specific statements regarding the effects of IGF1 and its distribution in relationship to activity and BDNF levels, further studies also exploring the relationship and dynamics of IGF1 with various carrier proteins would be intriguing and beneficial.

In order to investigate and interpret the correlation between cortisol and BDNF, it is important that the data correspond to the physiological norm. During stressful situations, significant short‐term increases in cortisol can be observed in healthy individuals (Cay et al., 2018). The measured diurnal profile of cortisol showed a morning peak and a decrease throughout the day, without reactive peaks, for all participants (Figure 6; *t*_10_), which corresponds to physiological dynamics (Krieger et al., 1971). Furthermore, it is reasonable to assume that all participants had healthy hormone regulation. Altered cortisol levels have been observed in the context of mental and neurodegenerative diseases (Csernansky et al., 2006; Fiksdal et al., 2019; Li et al., 2023). In the case of Altzheimer's disease, changes in the cortisol‐to‐BDNF ratio have been demonstrated (Curto et al., 2017). Animal experiments have shown lower levels of BDNF in response to high‐dose cortisol applications (Chao & McEwen, 1994; Schaaf et al., 2000), suggesting clear interactions between cortisol and BDNF levels. Studies have documented a similar dynamic for plasma BDNF to that for cortisol, with a steady decrease throughout the day (Begliuomini et al., 2008; Pluchino et al., 2009). However, the inferred assumption of co‐regulation was not confirmed in the present study. No short‐term effects or correlations could be derived, and the diurnal profile did not show a direct response of BDNF to the circadian pattern of cortisol in the periphery. A measurable influence on BDNF seems to be more associated with long‐term and pathological cortisol levels.

The role of BDNF as a biomarker and its utility for inter‐individual comparisons is challenged because of numerous individual influences and resulting responses. In summary, the results of this study indicate the following: (1) there is no circadian rhythmicity of plasma BDNF in young, healthy men; (2) there is a nocturnal decrease in serum BDNF during sleep in young, healthy men; (3) the type of sample collection (continuous peripheral indwelling cannula vs. immediate blood sampling) does not significantly affect the measured BDNF levels; (4) there is no significant correlation between cortisol and BDNF levels in serum or plasma; and (5) there is a moderate negative correlation between serum BDNF and serum IGF1. As a consequence of these findings, the use of BDNF for diagnostic purposes or as a biomarker is not limited by timing issues, and standardized protocols for sample collection and analyses do not necessarily need to consider the time of day.

A major limitation of the study is the small number of participants (*n* = 10). Furthermore, given that it was our goal to recruit a homogeneous test group of healthy young men, the results cannot be generalized to other populations, such as women or elderly people. Moreover, although studies indicate that measurements of BDNF levels in blood and plasma mirror BDNF levels in the brain (Klein et al., 2011), the analyses of blood samples can only reflect changes in the periphery, and we cannot provide information about central processes in the brain and their interactions.

For a validation and completion of the results, further studies with larger and more diverse samples are necessary. Additionally, intra‐individual measurements would be interesting to verify the collected data and, apart from assessing the quality of the methodology, to investigate possible differences in the context of altered light–dark cycles attributable to seasons or different activity levels. To explore the potential of BDNF as a biomarker further, standardized protocols for analytics are necessary. These protocols could establish a better comparability for generated readings and potentially identify a preferred source of BDNF. Further studies are also needed to assess whether the nocturnal decrease in serum BDNF is attributable to endogenous circadian rhythms or to a state of inactivity or sleep.

## AUTHOR CONTRIBUTIONS

Maren Ehrhardt, Notger G. Müller, Volkmar Leßmann and Patrick Müller designed the study. Maren Ehrhardt and Yves Duderstadt performed data collection. Maren Ehrhardt, Katrin Borucki, Tanja Brigadski and Volkmar Leßmann performed blood analysis. Maren Ehrhardt and Patrick Müller analysed the data and wrote most of the manuscript. Stefanie Schreiber, Yves Duderstadt, Notger G. Müller, Rüdiger Braun‐Dullaeus, Tanja Brigadski and Volkmar Leßmann made significant contributions to the design of the study and writing of the manuscript. Patrick Müller is responsible for research governance of the study. All authors approved the final version of the manuscript and agree to be accountable for all aspects of the work in ensuring that questions related to the accuracy or integrity of any part of the work are appropriately investigated and resolved. All persons designated as authors qualify for authorship, and all those who qualify for authorship are listed.

## CONFLICT OF INTEREST

None.

## Data Availability

Data that are not presented that support the findings of this study are available from the corresponding author, Maren Ehrhardt, upon reasonable request.
